# A Clinical Decision-Making Framework for the Use of Video Gaming as a Therapeutic Modality

**DOI:** 10.3389/fneur.2021.610095

**Published:** 2021-05-28

**Authors:** Debbie Espy, Ann Reinthal, Vanina Dal Bello-Haas

**Affiliations:** ^1^School of Health Sciences, Cleveland State University, Cleveland, OH, United States; ^2^School of Rehabilitation Science, McMaster University, Hamilton, ON, Canada

**Keywords:** virtual reality, exergame, motor learning, clinical decision making, clinical framework, exercise therapy, neurological rehabilitation

## Abstract

Virtual reality and video gaming offer modulation of more exercise and motor learning parameters simultaneously than other modalities; however, there is a demonstrated need for resources to facilitate their effective use clinically. This article presents a conceptual framework to guide clinical-decision making for the selection, adaptation, modulation, and progression of virtual reality or gaming when used as a therapeutic exercise modality, and two cases as exemplars. This framework was developed by adapting the steps of theory derivation, whereby concepts and parent theories are brought together to describe a new structure or phenomenon of interest. Specifically, motor learning theory, integrated motor control theory, Gentile's Taxonomy of Tasks, and therapeutic exercise principles were integrated to develop this framework. It incorporates person (body segment), environmental, and task demands; each demand is comprised of realm, category, choice, and continuum parameters as motor training considerations and alternatives for decision-making. This framework: (1) provides structure to guide clinical decisions for effective and safe use of virtual reality or gaming to meet therapeutic goals and requirements, (2) is a concise and organized method to identify, document, and track the therapeutic components of protocols and client progression over time; (3) can facilitate documentation for reimbursement and communication among clinicians; and, (4) structures student learning, and (5) informs research questions and methods.

## Introduction

Virtual reality (VR) is the use of computer hardware and software forming interactive simulations to present users with opportunities to engage in environments that feel and appear similar to real world events and objects (1). It is an increasingly accepted modality for physical and cognitive rehabilitation (2–4). The VR environment can be described as non-immersive (i.e., a screen - computer generated environment), semi-immersive (i.e., flight simulator or game with a mix of real and virtual interactive elements), or fully immersive (i.e., HCT Vive) based on the level of immersion and the extent of being present or part of the VR world; the higher level of immersion corresponds to a more realistic VR environment to the user (5). A key feature of VR is the active participation in the VR experience via control interface input into the computer system. As video games, serious games, and virtual environments present a virtual world that users can manipulate, they are technically, and often, included within the scope and definition of VR.

Commercial, off-the-shelf video games and gaming consoles (referred to as gaming in this paper) were initially developed for entertainment purposes, but share some of the same features and advantages of much more expensive, custom VR systems. Commercial games evolved as a means to encourage exercise in the general population (6, 7) and some of these have been adopted as therapeutic modalities for physical rehabilitation (8–10) because of their lower costs (11, 12). Reviews of the evidence of gaming as a therapeutic tool find effectiveness in a number of applications. For example, Chen and colleagues found that in people with Parkinson Disease the use of VR improved Berg Balance Scale (BBS) scores compared to other interventions (13). Similarly, significant improvements in BBS scores were found for VR interventions in people with chronic stroke (14). Laver et al. (3) determined the use of VR and interactive video gaming was not more beneficial than conventional therapy for improving upper limb function but suggested these modalities may be beneficial when used as an adjunct to usual care to increase overall therapy time. While these evidence summaries suggest there are real and potential benefits of VR, they also underscore equivocal conclusions, methodological issues (e.g., small sample size, rigor, quality), large variability in the protocols used (e.g., number of sessions, intervention duration, outcome measures), and the need for further research (15–18).

Gaming and VR are used in rehabilitation because they have several potential motor learning advantages over traditional exercise. They provide the massed motor practice and dosage (3) necessary to induce experience-dependent neuroplasticity (19–22). Multiple repetitions of task practice are essential for motor retraining (23–26) but repetitive practice of a single task is often boring for adults (8, 27, 28). Many individuals find gaming and VR more engaging and enjoyable than traditional exercise programs, thus are motivated to practice more (29, 30) and are less likely to withdraw from VR interventions (17).

The theory of flow highlights that a person's skills and the task demands should align, and that the intrinsic motivation for a task is best when the demands lie ideally along the orthogonal continua of anxiety to flow, and apathy/boredom to relaxation (31). Flow has been described as the optimal experience “when nothing else matters” (32) and conceptualizes dimensions that lead to these positive experiences and pleasurable mental states, such as balance between the skills of an individual and the activity's demands; merging of action and awareness; clear goals; immediate and unambiguous feedback; concentration on the task; perceived control over the activity; and intrinsic motivation toward an activity (autotelic) (32, 33). VR and gaming provide these experiences and the ability to modulate these dimensions.

Video gaming can provide a large range of task demands, allowing finer tuning of the challenge posed by a given intervention. Most significantly for neuro-rehabilitation, VR and active gaming provide rich opportunities for modulating the concurrent motor and cognitive demands of an activity to provide crucial dual- or multi-task therapeutic activities (34, 35). Likewise, VR may provide an enriched environment for problem solving and mastering new skills (35). Potential advantages for cognitive retraining among older adults (34, 36) and increased attention skills resulting from gaming have been reported (37, 38). A randomized controlled trial comparing physical exercise, cognitive exercise, and VR exercise demonstrated significant improvements in cognitive and physical function with VR exercise in older adults; VR exercise was also more favored than physical exercise (39). A recent review and a meta-analysis discussed positive effects of semi-immersive VR on cognition and physical function in people with mild cognitive impairment and dementia (40, 41).

Because VR and gaming are immersive (1, 42), they create a sense of engagement and presence (43), the sense of psychologically leaving the real location and feeling as if transported to a virtual environment for the users. The game's context may be more similar to an actual task, an important component of the ecological approach to cognitive-motor dual task situations (44, 45) which emphasizes that tasks should be as close as possible to real-world scenarios; virtual environments can simulate the crucial sensory cues of complex activities (46). All of these elements may account for the potential transfer of skills to comparable real-world activities (47), a concern in current practice (48).

Despite their advantages, VR and gaming are therapeutic modalities, not therapy in and of themselves. As such, the therapist must identify the specific goals that will be met through the use of gaming; and, gaming tasks need to be chosen to align with those goals and structured to provide the appropriate challenge (49). Performance needs to be monitored, outcomes evaluated, and learning achieved via gaming needs to be linked to the real-world context (49). Further, therapists need to ensure that gaming activities are safe, are not detrimental, and are cost effective.

Lack of time and information have been found to be the biggest barriers to incorporation of VR and gaming into rehab therapies (50–52), while therapist knowledge was found to be a prime facilitator (51). To this end, guidelines, frameworks and clinical practice recommendations are emerging. The “Kinect-ing” With Clinicians format was developed as knowledge translation for physical and occupational therapists integrating the Kinect system into practice (52). A framework has been developed to assist clinicians in choosing VR systems for pediatric patients in neurorehabilitation (53) and a practice guideline has been proposed for VR as an intervention (54). None of these addresses the clinical decision-making process in structuring and using the chosen games and platform though, particularly identifying critical therapeutic elements and their rationale.

### Purpose

Broadly, clinical decision-making frameworks guide and enhance the implementation of theory-based rehabilitation practice by providing a systematic approach to organize thinking, observations, and interpretations (55, 56). This paper describes a conceptual clinical decision-making framework and its utilization, through two cases as exemplars, in making and tracking decisions about the therapeutic elements of video gaming and VR modalities in clinical practice, particularly when used to address movement, mobility, balance, and motor relearning goals. Often the terms “framework,” “theory,” and “model” are used interchangeably, as are the terms “theoretical framework” and “conceptual framework.” We have purposefully chosen the term conceptual framework. A conceptual framework explains, graphically or in narrative form, one or more formal theories, in part or whole; as well as key factors, concepts, variables, and empirical findings from the literature to show relationships among ideas (57, 58).

### Framework Development

The need for the framework grew out of our laboratory and clinical studies researching off-the-shelf video gaming as a therapeutic tool in balance training and motor relearning for older adults and people post-stroke. We recognized that there are many potential motor-control and learning variables that can be modulated simultaneously with gaming and VR, as well as therapeutic exercise and neuro rehabilitation principles that must be appropriately considered. Our thought was to organize these elements and considerations into a framework that would facilitate clinical decision-making through making explicit the motor control, motor learning, and therapeutic exercise constructs accessible through VR and gaming-based therapy.

Walker and Avant's Theory Derivation (59) procedures were adapted to organize related concepts in a structural manner to illustrate these relationships as a framework. Theory derivation is an iterative process that considers theory and knowledge of the literature within an area of interest to explain possible new concepts and structures. Relevant concepts and structures are borrowed, modified, and redefined from a parent theory, in whole or in part, to explain a phenomenon of interest (59, 60). A theory derivation approach has been used in a wide range of health care literature to develop theories and to adapt existing theories, models, and frameworks (60–62).

We used the steps of Theory Derivation to provide systematic structure to the framework development. Basic steps include: (1) become familiar with the literature on the phenomenon of interest; (2) examine the literature of other applicable fields; (3) choose a parent theory to explain the phenomenon of interest; (4) identify concepts, components, and content from the parent theory to be used; and (5) modify, redefine or refine concepts, components and content from the parent theory (59). Our goal was not to develop a new theory or to adapt a theory, but rather, we developed a conceptual framework to organize and make explicit the therapeutic elements, principles, and considerations that underlie the use of VR and gaming as a motor rehabilitation modality.

**Figure 1:**
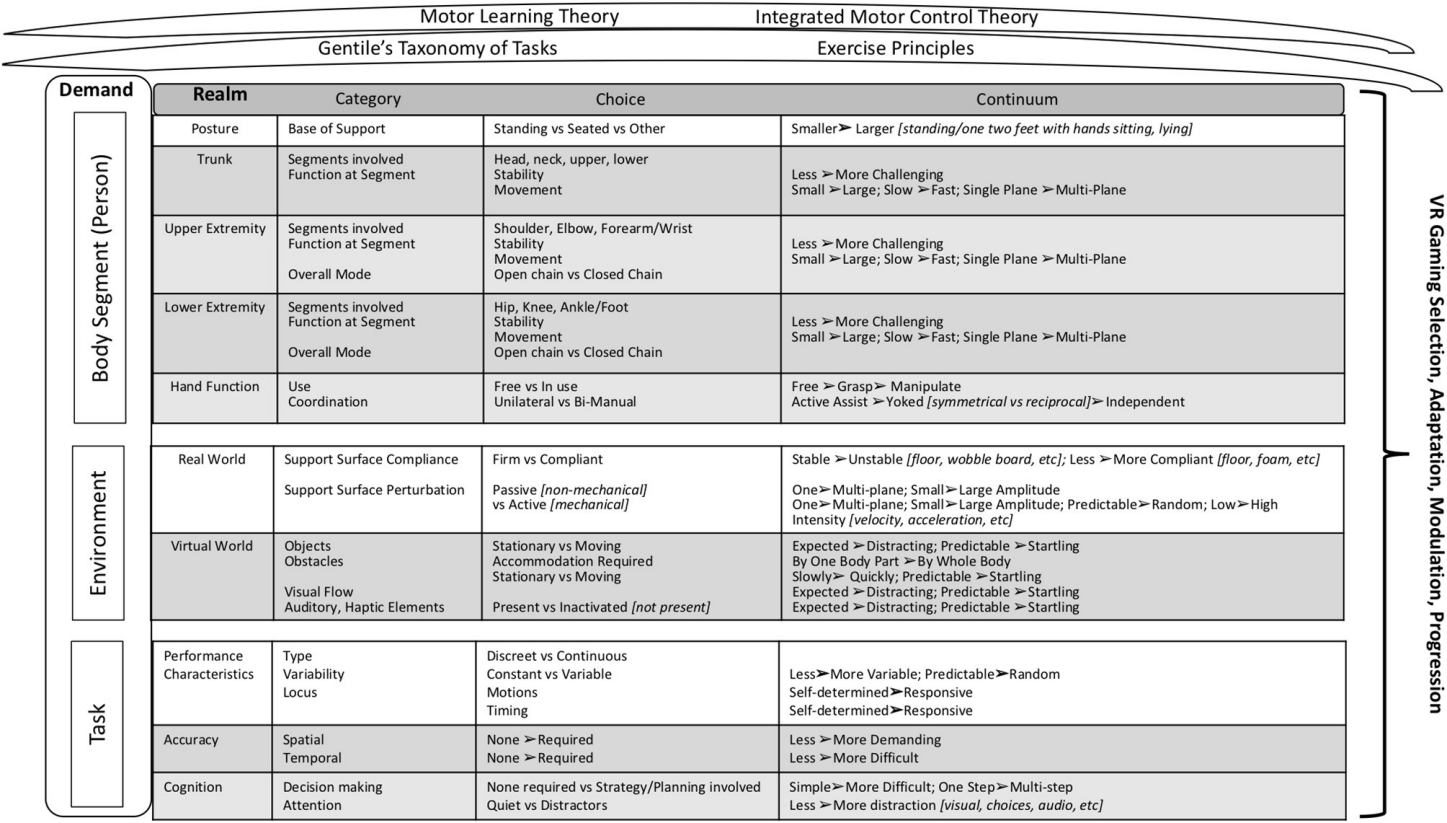
Clinical Decision Making Framework. The framework synthesizes motor learning and control and exercise science considerations as encountered in the use of VR or gaming technologies as a therapeutic training modality in rehabilitation. It is portioned into demands based on the person (body segment), the environment, and the tasks. The realms specify the potential areas to consider within each type of demand; and the category breaks down each realm into the motor learning, motor control, or exercise-based elements presented within that realm. Within each category, the difficulty of the motor control, motor learning, or exercise element presented can be modulated through discreet choices (Choice) and/or by increasing or decreasing the parameters indicated within “Continuum”.

Parent theories were carefully examined, and applicable components were extrapolated, and a wide range of literature was utilized for initial framework development, as described in the following section. Drawing on theory, concepts, principles and evidence, we organized these various elements and considerations into an initial framework for therapeutic game selection, adaptation, modulation and progression. The initial framework underwent an iterative process of review and refinement. For example, the framework was applied with 78 individuals, across ages, participating in various research and clinical studies in the laboratory of two of the authors (DE, AR), as well as in the clinical practice of all authors. Iterative application of the framework in this manner was used to refine included concepts and clinical utility such as ease of use, usefulness, acceptability, benefits, meaning, and relevance of the framework (Figure 1 for overview of the framework).

#### Parent Theories

In adapting the Theory Derivation Process to this framework development, the parent theories and concepts chosen included: motor learning principles, integrated motor control theory, and basic therapeutic exercise principles, as well as more specific concepts used in neuro rehabilitation. These theories and principles were chosen for this framework because they underpin motor training in neuro rehabilitation.

Motor learning refers to a set of internal processes associated with practice or experience that lead to relatively permanent changes in motor behavior (63). Retention of a learned task or skill is important as permanent changes are the desired outcome. Additionally, transfer of training (63), the ability of the client to draw on past experience to perform a new task or skill, are affected by practice conditions (63). Training parameters that impact retention and/or transfer of skills include repetition, time on task, type and schedule of feedback, locus of attention, context, and variability of practice. Variability in practice is most beneficial for retention and transfer of a motor skill (64).

Integrated motor-control theory conceptualizes movement as a product of the interaction among the *individual*, the *task*, and the *environment* (65), and incorporates many of the concepts of other systems-based theories [i.e., Dynamic Systems Theory (66)] in which movement is thought to be generated by an individual to meet the demands of a specific task performed within a specific environment. Individual, task and environment attributes contribute to the execution of movement tasks. According to Shumway-Cook and Woollacott (65) *individual attributes* may involve action (e.g., motor system, impairments), perception (e.g., factors that affect or limit the internal registration or integration of sensory information), and cognition (e.g., factors such as attention, emotions, motivation, ability to attend to environmental stimuli during the execution of tasks or activities). *Task attributes* define and constrain the execution of a movement task, and are classified into a discrete task with a discernable beginning and ending point (e.g., sit to stand) or a continuous task with a variable ending point (e.g., walking). Whether the base of support (BOS) is stationary or changing is an additional task attribute; and, task considerations include upper extremity (U/E) manipulation requirements, the amount of attention demanded by a task, and the variability of the movement itself. *Environmental attributes* can be divided into regulatory (i.e., factors that shape the movement) and non-regulatory conditions (i.e., factors that may affect performance but do not directly shape the movement, such as background noise or air temperature). In a stationary environment, the regulatory conditions involve a fixed terrain and non-moving objects, and the environment influences only the spatial parameters of the movement. When activities occur in a moving environment, where objects, other people, or the supporting surface are in motion, movements must conform to both spatial and temporal parameters of the environment.

Gentile's Taxonomy of Tasks (67) expands on aspects of the above task and environmental demands and creates a classification for movement activities based on these. The least challenging tasks are those that do not require U/E manipulation and that are performed in a stationary, non-variable environment. Tasks in the taxonomy steadily become more difficult with the addition of mobility requirements, a manipulation component, or increased variability in the environment. The degree of difficulty associated with a movement task and progressing the complexity of a task are determined by changing its taxonomy. When temporal environmental factors are stationary, only the spatial factor of the movement is controlled by the environment—tasks in which temporal environmental factors remain stationary and fixed from trial to trial are termed *closed tasks*. Tasks in which the temporal factors of the environment are stationary but the spatial factors of the task, such as the size or location of objects, vary from trial to trial, are called *variable motionless tasks*. When environmental factors include objects or persons that are moving, both spatial and temporal factors of the movement are determined by the environment. Tasks in which these environmental factors change from trial to trial are termed *open tasks*.

Rehabilitation, especially neuro rehabilitation, largely involves structuring practice to facilitate acquisition or re-learning of skills, with attention to all of the considerations inherent in the above parent theories. Other relevant concepts and principles that underpin therapeutic exercise in neuro rehabilitation include open chain vs. closed chain modes of exercise, considerations of the stages of motor learning, and categories of motor skills including dual-task skills (64).

#### Framework Structure

In analyzing our use of gaming, the games themselves, and our clinical thought processes in using gaming in therapy, we created an initial structure for the framework around the integrated motor-control theory concept of movement as a product of the interaction among the individual, the task, and the environment (65). As such, our framework is comprised of three types of **demands**: person (body segment), environment, and task (Figure 1). Each demand is comprised of one or more **realms**, domains of interest within that demand type. The ***body segment demands***are person-level and define the postural stability, movements, and necessary interactions between these two, required for successful game play. These are analyzed by body segment **realms**, specifically *posture, trunk, U/E, lower extremity (L/E)*, and *hand function*. The ***environmental demands***include the external characteristics of the *real-world* **realm** in which the player (person) is gaming, as well as the environmental context provided by the *virtual* (game) **realm**. The ***task demands***characterize additional motor learning and motor control constraints and affordances related to the specific game, such as attentional and cognitive requirements. Each **realm** is comprised of **categories**, specific factors to consider that are reflective of the **realm**. Within a **category**, a judgement is rendered as to the relative difficulty of the item as presented in the specific person/game/set-up under consideration. In some cases, it is a **choice** (yes/no, present or not); in others it is a **continuum** (from less to more difficult or complex).

### Framework–Theory Integration

#### Person/Body Segment Demands

Gaming and VR platforms dictate, to a greater or lesser degree, the functional requirements of a game, for example, avatar-based systems often require full body participation, while accelerometer or inertial measurement unit (IMU) based systems can respond to single segment motion, allowing but not requiring full body motion. The functional requirements of a game that intersect with the individual's attributes include overall posture, BOS, and specific combinations of joints providing stability or movement. While some games can be played, for example, in sitting or standing, others dictate one particular posture. Base of support can be dictated by certain games (e.g., kicking in single limb stance), but others do not respond to foot placement at all, allowing any stance for play. Certain games and platforms allow or can be adapted to be played from sitting or with one or both upper extremities in weight bearing.

*Stability* and *mobility* are categories of motor skills: stability involves maintenance of a posture at rest or during movement, and mobility involves controlled movement of the body or segment from one posture to another (64). In the given posture, with the given BOS, the activity may engage the full body or any segments in stability or mobility motor skills. Segmental analysis reveals whether a segment is stable or mobile, allowing for both therapeutic emphasis and avoidance. The lower extremities (or single leg) in stance must provide stability and the trunk may be required to provide a stable base for arm motions. In contrast, one lower extremity may be in motion (kicking) while the other provides stable support, or the trunk may move with the arms to complete the activity (e.g., trunk rotation with arm swing in a racket sport). Segments can be required to provide stability and movement simultaneously. Mobility is further graded by its amplitude or arc of motion, the speed of movement, and the planes of motion involved in the game. Extremities can be used in both open and closed chain fashions, with some modifications to the experience often needed for closed-chain upper extremity use. Open chain motion involves a freely moving distal segment while, in closed chain motion, the distal segment is fixed (64). Modulating this parameter allows some specificity of training to the eventual, real world tasks of interest.

With some platforms and games, hand use may be minimal to unnecessary, while others require it. Accelerometer-based games typically require enough dexterity to manage the controller. Cases in which hand use is a therapeutic goal may require adding various manipulanda, which are included in certain games and can be easily adapted by the clinician in others. Hand function required by a game to stabilize, grasp, or manipulate the controller and object can be bilateral or unilateral, and can often be done in a variety of active assisted fashions. Finally, some games require elements of symmetrical or reciprocal arm activity, such as swinging a golf club, while others require independent left vs. right hand use, such as playing a guitar.

#### Environmental Demands

Active games and VR create virtual environments that must be considered along with the real-world environmental constraints of any activity. There are no real consequences if the constraints of the virtual environment are not met, though they may be felt as “real” by players engaged in the game. The real-world environment, however, can present real constraints. Thus, the model delineates real and virtual environmental constraints.

Aligning with Gentile's taxonomy (67), the framework identifies whether the real environment is stable or in motion, whether this varies between trials, and if the variability is predictable. Specifically, the weight bearing surface can be firm or compliant on a continuum of being more stable (less difficult), such as standing on the floor, to less stable (more difficult), such as standing on a Bosu ball. Induced perturbations can be caused by a passive surface, such as a wobble board, or by an active mechanical surface, such as a motorized platform. Surface perturbations can also be: unidirectional (e.g., wobble board) or multidirectional (e.g., Bosu ball), smaller to larger amplitude, lower to higher intensity (velocity/acceleration), and predictable vs. random.

The framework classifies the virtual environment into four broad categories: objects, obstacles, visual flow, and auditory or haptic components. There are objects in the virtual world (visual field) that do not need to be accommodated; these are one type of non-regulatory conditions (67). These objects can be stationary or moving, related to the task (expected) or unrelated (distractors) that must be ignored, and predictable or startling. For the virtual obstacles on the other hand, accommodation by one body part or by the whole body (regulatory) is an aspect of the game. These can be stationary (relative to the overall motion of the virtual environment) or in motion (moving distinctly from the rest of the virtual environment), and they can move slowly to quickly and predictably to unpredictably. A visual flow is created in the virtual environment giving the sense of moving directionally, including looming or receding as appropriate (68). This flow can be as expected (i.e., matches the movement of the player/avatar appropriately) or unexpected (mis-matched) and predictable or startling. Finally, auditory or haptic elements can be active or de-activated, and when active, can range from expected to distracting and from predictable to startling.

#### Task Demands

The ability to modulate motor learning and performance characteristics are an advantage of gaming over standard modalities. Game tasks may be discrete or continuous. Consistent with motor learning theory, discrete tasks provide breaks for rest, hypothesis testing, feedback, or attention (63). Continuous tasks demand more endurance (motor, attentional, cardiopulmonary, et cetera), and potentially more automaticity in task performance, consistent with more mature stages of motor learning (64). They also provide more overall practice repetitions and the higher dosage necessary for motor retraining (23–26). Tasks may also be variable or not, as described in Gentiles's taxonomy (67); if variable, they are graded as minimal to extensive and predictable to random. In the early stages of learning, blocked practice enhances motor learning, while retention is better in later stages of learning with random practice (63). The contextual interference introduced with more variability enhances retention, learning, and likely transfer, but must be adjusted to the skill level of the learner (69).

Timing and motion responsiveness range from self-determined, in which the player chooses the specifics of the timing and motions, to responsive, in which the game dictates all of these parameters. Therapeutically, both modes demand motor planning and initiation, while a responsive mode also promotes perseverance, endurance, faster responses, and automaticity – corresponding to progression through stages of motor learning (64). More impaired individuals may master game play more easily when both timing and motions are self-determined because they can slow the pace and are allowed more motion options. Games may have no accuracy demands or may require a high degree of spatial and/or temporal accuracy. Both of these are components of Gentile's taxonomy and can be regulatory (essential to meet) or non-regulatory (67). When temporal and spatial accuracy demands are simultaneous, one component typically predominates (63).

The requirement to maintain specific postures and motions while also attending to a game mean that gaming and VR are inherently dual tasks, the most challenging category of motor skills (64). A range of motor and/or cognitive elements can be added, or their difficulty modulated through game choice. The cognitive domain includes decision-making requirements, and attentional demands (70). Decision-making demands may be none to simple choices (e.g., to swing or not at a pitched baseball) to multiple choices (e.g., best route to avoid an obstacle in a racing game.) to requiring multi-step strategic planning for successful play. Finally, games have few to many distractors (non-regulatory elements) which may or may not be relevant to the game and, if present, are an additional decision-making requirement (i.e., to be ignored or attended to).

## Discussion

This framework has been developed for use by rehabilitation clinicians working with clients with mobility, movement, and motor re-learning goals. It is designed for VR or gaming-based movement and practice based therapeutic exercise, particularly motor learning and control. It is useful for games that present and respond to full or large body motions, and it is not technology specific. The framework can be applied across gaming and VR platforms, including newly developing technologies, because it is non-system specific in its design and terminology and it incorporates well-established theoretical concepts and principles. It is, however, a *motor* rehabilitation framework and is not designed to be used with games that emphasize cognition or strategy, especially those played primarily via a joystick, buttons, keyboard, et cetera. It also does not address gaming targeted at fitness, or primarily cardiovascular/pulmonary interventions.

### Framework Use

In every therapeutic activity, the clinician must intentionally prioritize the critical active ingredients to address or to avoid, including body segments and movements, correct levels of challenge, and specifics about the physical environment. Gaming has significantly more elements to consider than typical therapeutic activities; virtual features can provide additional challenge, level and type of task constraints, and motor learning and control components that should be emphasized. The clinician must choose and tailor the game effectively to align the therapeutic aspects with the client's treatment goals. The framework guides the clinician in considering all of the critical variables, specifically in the context of gaming. It also facilitates evaluation of gaming-specific factors that might not be an issue in a non-gaming context, for example, overload of cognitive demands, startling elements, and/or additional virtual obstacles to accommodate.

It is important to be able to modulate the difficulty of any therapeutic exercise or activity. In gaming, adding or removing motor control elements increases or decreases the difficulty of the activity. Likewise, within categories, adding or eliminating the yes/no elements increases or decreases the active therapeutic elements impacting the player (client), as does moving toward the harder or easier end of the continuum in graded elements. For example, to increase the challenge of an activity, temporal or spatial accuracy demands can be added then increased, expected movement excursions can be increased, and dictated base of support can be decreased. It should be noted that moving through the game options or levels may increase or decrease the difficulty and those differences can be identified through the framework.

The framework guides decisions about modifications to the real or virtual environment or task. Game analysis, through the framework, identifies elements that the client may not be able to tolerate, or which may be detrimental, for example a game may be overly challenging or specific gaming variables may interfere with the therapeutic session. Some aspects may be contraindicated or unsafe for an individual, such as altered visual flow (Realm–Virtual World, Category–Visual Flow) or stepping requirements (Realm–Lower Extremity, Category–Function at each Segment, Choice–Movement). Analysis through the framework can point to needed real or virtual modifications, such as instructing the client to ignore certain obstacles, turning off the sound, or changing the support surface from standing to sitting.

#### Context in Which It Is Useful

Clinicians must document the therapeutic interventions that clients experience, not the games they play. The framework helps to articulate (document) the segments involved, the movement or stability requirements of the activity, the concurrent cognitive demands, and the other therapeutic elements of the treatment session. This then facilitates recording the added elements or progression within elements as well as any areas being avoided and the reasons. This treatment documentation is important for communication among providers, reimbursement, and to note and guide progress toward goals. Likewise, in gaming or VR research, this structure facilitates protocol design for and documentation of the impactful elements and levels of a gaming intervention, which allows accurate investigation and comparisons of gaming-based interventions. In the educational setting, the framework can help students identify and articulate the many considerations behind the use of gaming as a therapeutic modality and offers a structure for examining the theory-based therapeutic elements. Below are two examples illustrating use of the framework in two settings, a clinical research study and a clinic.

#### Case #1: Research Application

The framework was used to develop a gaming progression algorithm for a randomized controlled trial examining intense harnessed balance training for individuals post stroke. Five categories of **Person demands** represented the variety of standing/stepping balance challenges encountered during typical daily mobility activities: (1) anterior posterior stepping (AP stepping); (2) medial lateral stepping (ML stepping); (3) feet in place AP/ML/vertical center of mass (COM) weight shift (weight shift); (4) feet in place trunk turning (rotation); and (5) alternating single leg stance with dynamic kicking (SLS). A four-level progression of Kinect (Microsoft) video games combined with varied standing support surfaces was used to gradually increase the intensity demand within each balance activity category, systematically maintaining high intensity practice demands.

In the **Person demand** ***Lower Extremity Realm***, the framework was followed to vary activities in *Overall Mode* and *Function at Each Segment* from closed chain with stability demands (weight shift and rotation), open chain with mobility demands (ML and AP stepping), or a combination of both (SLS, with closed chain SLS and open chain LE kicking). In the ***Posture realm***, the games were chosen to vary in terms of smaller to larger *COM* control demands (rotation → weight shift → stepping → SLS). The ***Upper Extremity realm***was varied in the need for open chain shoulder and elbow activity being required or not required in a given game.

The ML stepping progression illustrates how the framework guided **Environment** and **Task demand** modulation to increase training intensity using two games. In the first game, 20,000 Leaks (Leaks), the player had to plug a varying number and placement of leaks in a surrounding aquarium. In the second game, Reflex Ridge (RR), the gamer rode a moving mining cart while dodging various obstacles. In the **Environment**, all ***realms***were varied. In the ***Real World realm*, **the *support surface* was changed by moving from solid (floor) to compliant (gym mats) surfaces. In the ***Virtual World realm***, the *objects* were moving, distracting, and unpredictable in both games, however RR demanded speedy, full-body accommodation for random *obstacles*. RR also added managing *visual flow* while riding the moving cart. The **Task** was varied across three r**ealms**. For the ***Performance characteristics***, the *locus* was self-initiated in Leaks and responsive in RR, and *spatial* **accuracy** only was required in Leaks while both *temporal and spatial* ***accuracy***were needed in RR. ***Cognitively***, both games required *decision-making* and *attention*, but the demands of RR were more complex and required speed.

#### Case #2: Clinical Application

In the clinical case example, the therapist and client with left hemiplegia first established a collaborative treatment goal: reaching the left arm to a table top in sitting with less than 30 degrees of elbow flexion in order to stabilize objects for various bimanual tasks. This was currently effortful and accomplished very slowly, with shoulder abduction and 90 degrees of elbow flexion. The critical **Person** component was in the ***Upper Extremity realm***, with the *Segments, Function*, and *Overall Mode* requiring open chain shoulder and elbow movement. The goal defined the ***Posture realm***as seated and the ***Lower Extremity***and ***Hand Function realms***were deemed not essential, however rotation *movement* of all *segments* of the ***Trunk realm***was desired. A racket type game was chosen but modified for play in sitting and for holding the racket with both hands. This “yoked” reciprocal pattern was chosen to decrease left arm task intensity, with the right arm/hand actively assisting the left during dynamic racket swinging. A variety of game options existed using the Wii and Kinect games of baseball batting practice, tennis/table tennis, and golf. While tennis/table tennis involved both a forehand and backhand swing, both of which were desired movement practice motions, golf and batting could be varied similarly by requiring a right vs. left-handed swing, so **Environment** and **Task demands** were considered next in determining initial game choice.

The **Environment** was least critical in this case; to keep the task at an optimum level of difficulty for this client, no ***Real World realm****support surface compliance* or *perturbation* was desired and no ***Virtual World realm****obstacles, visual flow*, or *haptic/auditory elements* were selected since it was known that distractors made movement very difficult for this individual. The ability of the client to manage *moving objects*, however, needed to be assessed along with additional **Task demands** in all ***realms***. In the ***Performance Characteristics realm*, **tennis/table tennis were a more *continuous type* than the *discreet* batting practice or golf swing. Golf had the least *variability* and was the only *self-determined locus*. It required *spatial*, not *temporal* ***Accuracy***, while the *spatial accuracy* in batting practice was less difficult (less amplitude and variability) than tennis/table tennis. Finally, in the ***Cognitive realm***, golf required the least *decision-making* and *attention*, and tennis/table tennis the most. Since this client was initially able to manage the *spatial/temporal* ***accuracy***demands of batting but not tennis/table tennis, practice began with batting and later progressed to table tennis. In addition, **Environment** ***Virtual World****auditory distractor elements* were later added to better meet the client's goal of movement performance in noisy environments.

### Limitations and Conclusions

It must be noted that this framework does not address feedback explicitly. Feedback is a crucial and complex component of motor learning (63, 64). Video games provide greater or lesser degrees of feedback about current play (knowledge of performance) through the avatar and visuals of the game itself. They also provide knowledge of results through points, scores, sounds, and visuals of cheering crowds, et cetera. This feedback is of variable scheduling, accuracy, and utility, depending on the platform and the game. While these both may be utilized at the clinician's discretion, the clinician should be providing feedback specific to the therapeutic tasks or movements, not to the game play. This is especially important for transfer or generalizability of the motor learning to real world conditions. Finally, this framework does not explicitly guide the process of taking the skills mastered in the gaming environment into real-world contexts. As with any motor learning intervention, the clinician must structure treatment sessions to include practice ultimately in real world contexts, out of the therapeutic environment (71). This framework does facilitate explicit identification of the therapeutic components involved in the gaming intervention which will need to be matched to the necessary real-world activities, practiced in the gaming environment, then practiced and assessed in the real-world environment.

Frameworks exist for many aspects of clinical decision-making within rehabilitation: for types of treatment (72, 73), for aspects of practice, such as goals and content of exercise interventions (74); or for decision-making (55, 75). Holden (47) noted the need for designers and users of VR in rehabilitation to know VR technology *and* motor learning principles, and to match VR features to those principles. This framework facilitates this necessary clinical decision making with the client's needs and goals foremost.

## Data Availability Statement

The original contributions generated for the study are included in the article/supplementary material, further inquiries can be directed to the corresponding author/s.

## Author Contributions

All three authors contributed to this conceptualization/idea of the Framework as well as the research and model development. DE and AR completed iterative testing of the model. All authors assisted in writing the manuscript.

## Conflict of Interest

The authors declare that the research was conducted in the absence of any commercial or financial relationships that could be construed as a potential conflict of interest.
